# Case Report: Liquid-based cytology diagnosis of pulmonary mucormycosis

**DOI:** 10.3389/fmed.2025.1590011

**Published:** 2025-07-23

**Authors:** Shuai Luo, Xiaoxue Tian, Ting Xu, Yao Li, Qing Ji, Jinjing Wang

**Affiliations:** Department of Pathology, Affiliated Hospital of Zunyi Medical University, Zunyi, Guizhou, China

**Keywords:** cytology, liquid based cytology, mucormycosis, diagnosis, differential diagnosis

## Abstract

**Background:**

Mucormycosis is an infrequent yet life-threatening opportunistic fungal infection, typically secondary to diabetic ketoacidosis. Owing to its non-specific clinical presentation and auxiliary test findings, timely diagnosis remains difficult. While fungal culture, direct smear, histopathological biopsy, and molecular diagnostic techniques are available, cytological evaluation and histopathological biopsy remain the principal modalities in clinical settings. Notably, cytological identification of mucorales remains rare.

**Case presentation:**

A 41-year-old male presented with a 20-day history of productive cough and fever, accompanied by a recent onset of hemoptysis. His medical history included type 2 diabetes mellitus. Diagnostic confirmation of mucormycosis was achieved through conventional cytological smear, liquid-based cytology, cell block preparation, tissue biopsy, and specialized staining techniques. Pulmonary mucormycosis was ultimately diagnosed.

**Conclusion:**

This report documents a case of pulmonary mucormycosis identified via conventional cytological smear, liquid-based cytology, and cell block preparation. Liquid-based cytology and cell block techniques offered a non-invasive and more expedient approach, highlighting their value in the early identification and intervention of mucormycosis.

## Background

Mucormycosis is a rare and life-threatening opportunistic fungal infection that commonly arises as a complication of diabetic ketoacidosis (1). With a case fatality rate of 70%–90% (2), timely diagnosis and intervention remain essential. Nevertheless, its non-specific clinical manifestations and limited diagnostic specificity of auxiliary examinations often impede early identification.

This case report documents the successful diagnosis of pulmonary mucormycosis using conventional cytology smear, liquid-based cytology, and cell wax block techniques. Notably, liquid-based cytology yielded a higher diagnostic yield by providing a cleaner background and clearer three-dimensional cytopathological features. Recognizing the distinct morphological characteristics of hyphal elements across cytologic modalities, alongside a thorough understanding of their cytomorphology, enabled precise diagnosis and informed therapeutic decisions, thereby contributing to improved clinical outcomes.

## Case presentation

On October 8, 2023, a 41-year-old male patient was hospitalized with a 20-day history of cough and expectoration accompanied by fever, which had acutely worsened with hemoptysis 1 day prior. His medical history included type 2 diabetes mellitus. The illness was preceded by an upper respiratory tract infection, after which a paroxysmal cough developed, producing small amounts of white sputum and intermittent fever, peaking at approximately 40°C. Despite receiving empirical anti-infective therapy at a local facility, symptoms persisted without notable relief. Hemoptysis, amounting to approximately 100 ml of blood without clot formation, occurred abruptly and spontaneously the day before admission. The patient denied chest pain, fatigue, anorexia, dizziness, headache, palpitations, chest discomfort, dyspnea, sore throat, nasal congestion, or rhinorrhea. He presented to the emergency department for evaluation of hemoptysis. No changes were reported in appetite, sleep, urination, bowel habits, or body weight.

**FIGURE 1:**
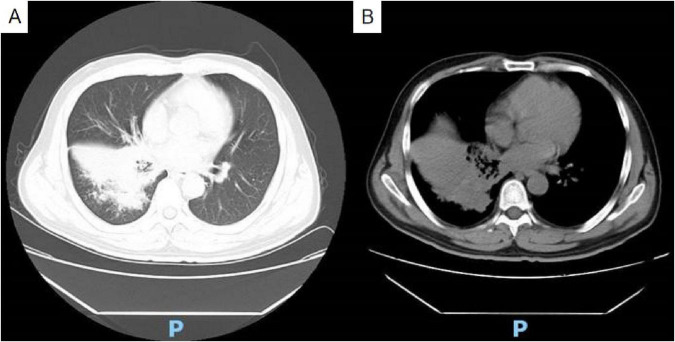
Non-contrast chest CT reveals bilateral pneumonia, segmental consolidation, and bronchial stenosis in the lower lobe of the right lung **(A)** lung window; **(B)** soft tissue window.

Chest contrast-enhanced computed tomography (CT) upon admission revealed bilateral pneumonia, segmental consolidation, and bronchial narrowing in the right lower lobe, with no radiological evidence of malignancy (Figure 1). Follow-up anti-inflammatory assessment was advised. Mild mediastinal and right hilar lymphadenopathy was observed, and attenuation in the right sixth rib appeared heterogeneously distributed.

The patient received symptomatic hemostatic and anti-infective interventions for right-sided pneumonia accompanied by hemoptysis. Electronic bronchoscopy revealed tenacious white secretions within the right bronchial lumen and a newly identified lesion at the bronchial orifice of the right lower lobe. The lesion exhibited marked vascularization, active hemorrhage, and extensive white necrosis, resulting in complete occlusion of the bronchial opening. The surrounding mucosa demonstrated marked hyperemia and edema, while distal bronchial structure was obscured due to necrotic obstruction and associated luminal expansion. Similar inflammatory changes were noted in the mucosa of the middle lobe. These findings raised suspicion for a malignant neoplasm with associated bionecrosis at the right lower lobe bronchial entrance. Diagnostic procedures included bronchial brushing, cytological analysis of bronchoalveolar lavage fluid, cell block preparation, and bronchial tissue biopsy.

**FIGURE 2:**
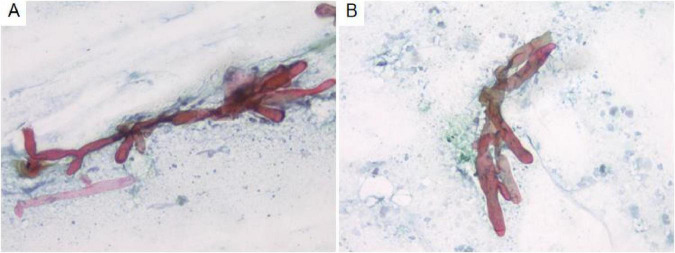
Cytological analysis of bronchial brush smears demonstrates irregular filamentous structures **(A)**. These filaments are broad, reddish, band-like, and resemble kelp, with branching angles approximating 90° **(B)**. Pap staining, ×400.

Cytological examination of the bronchial brush smear revealed irregular, filamentous structures on pap-stained slides, characterized by broad, red, ribbon-like formations exhibiting folds, undulating contours, and kelp-like morphology. These structures lacked septation and demonstrated branching at approximately 90° angles (Figure 2).

**FIGURE 3:**
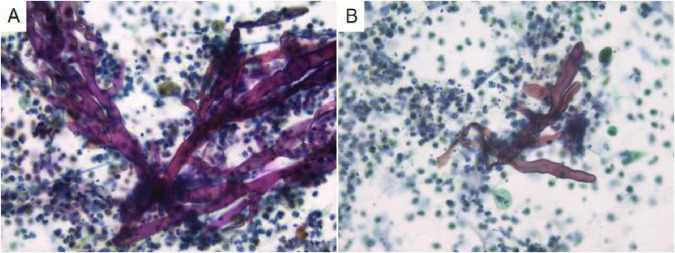
In alveolar lavage cytology, amidst inflammatory and necrotic cellular debris, thick, irregular filaments **(A)** are observed. These structures are commonly twisted, folded, and exhibit irregular branches in 90° angles **(B)**. Liquid-based cytology, Pap staining, ×400.

Analysis of the alveolar lavage fluid identified a substantial presence of inflammatory cells and necrotic debris, accompanied by irregular filamentous formations. These filaments appeared thicker, more twisted, and folded, with inconsistent branching and frequent right-angle bifurcations (Figure 3).

**FIGURE 4:**
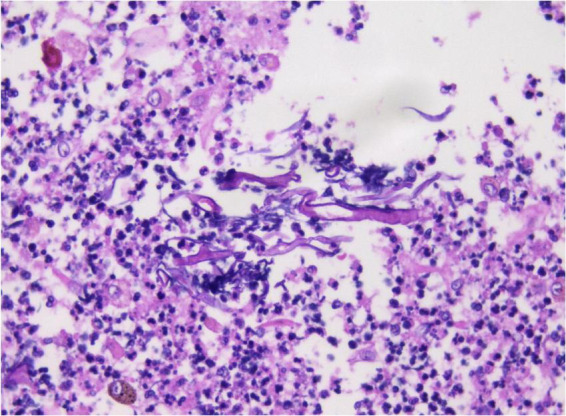
Cytology wax blocks clearly display irregular filamentous structures. H&E staining, ×400.

Given the abundant cellular content in the alveolar lavage fluid, a cytological paraffin block was constructed and subjected to hematoxylin and eosin (HE) staining (Figure 4). Within the necroinflammatory background, numerous filamentous hyphae were evident, either dispersed or aggregated. At high magnification, the hyphae were observed as broad, thick, folded, and corrugated structures, with disorganized structure and infrequent branching. Occasional filaments extended laterally at right angles from the main axis.

**FIGURE 5:**
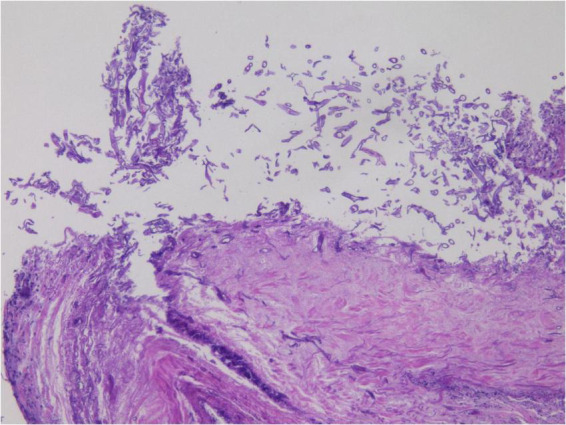
Hematoxylin and eosin (HE) staining in small tissue biopsy, with large amount of hyphae in the necrotic area under low magnification. H&E, ×200.

**FIGURE 6:**
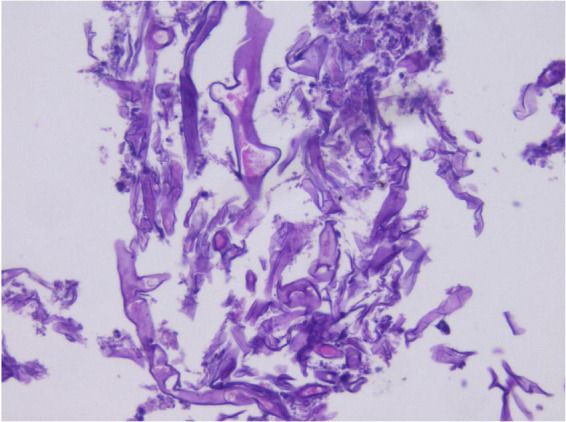
High-magnification imaging reveals folded, wrinkled, or swollen tubular. H&E, ×400.

**FIGURE 7:**
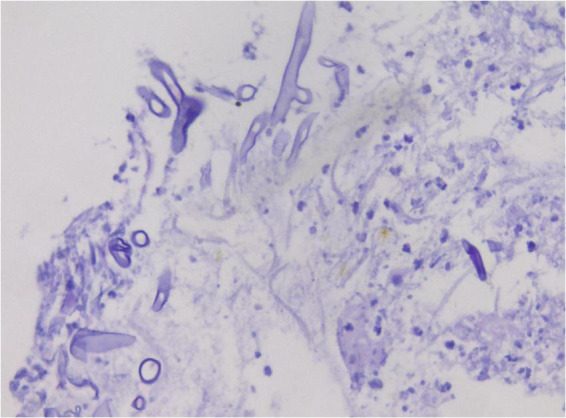
The Schiff (PAS) staining shows the mycelium of the pink bacterial wall. ×400.

**FIGURE 8:**
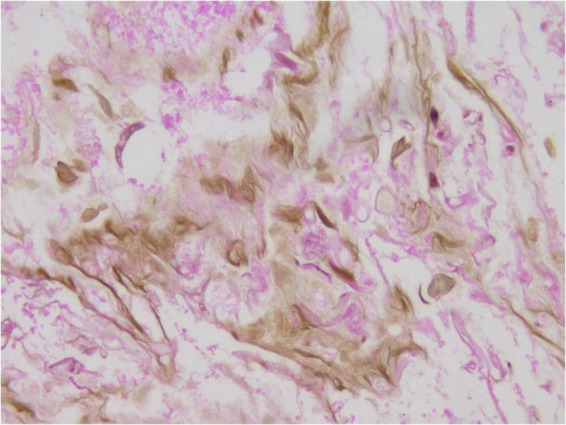
Grocott staining shows the hyphae of the-black wall. ×400.

In the limited bronchoscopic tissue biopsy, low-power HE staining revealed extensive inflammatory infiltration and necrosis within the bronchial mucosa (Figure 5). In certain regions, the pseudostratified ciliated columnar epithelium exhibited necrosis and detachment, accompanied by abundant hyphal elements in the necrotic zones. High-power examination demonstrated wide, tubular hyphae (approximately 10–15 μm in diameter), displaying irregular contours including folding, wrinkling, or swelling (Figure 6). These disorganized hyphae showed minimal branching, with occasional right-angle offshoots emerging laterally. In contrast, some hyphae manifested as unbranched cystic forms with transverse circular, elliptical, or polygonal profiles. Periodic acid–Schiff (PAS) staining delineated pink-stained mycelial walls (Figure 7), while Grocott methenamine silver (GMS) staining highlighted hyphae with distinct brown-black cell walls (Figure 8).

A comprehensive evaluation integrating traditional cytological smear, liquid-based cytology, cell block preparation, and histopathological biopsy confirmed the presence of broad, tubular hyphae, establishing a definitive diagnosis of pulmonary mucormycosis.

The diagnosis of mucormycosis in the right lower lung was confirmed on October 12, 2023. Initial management included antifungal therapy, glycemic control, antitussive treatment, and other supportive measures, followed by administration of a pituitary intravenous pump and two cycles of amphotericin B (5 mg, q6h). Despite this regimen, the patient developed a high-grade fever, and subsequent blood cultures identified *Citrobacter freundii*, prompting the initiation of imipenem at 1 g every 8 h. Given the persistent pulmonary infection and hemoptysis, surgical intervention was evaluated; however, elevated surgical risk due to ongoing bacteremia and suboptimal glycemic control precluded operative management. As a result, antifungal and antibacterial therapies were maintained in parallel with intensive glycemic regulation.

Clinical status subsequently stabilized. The patient was discharged on October 18 and monitored over an 8-months follow-up period. No evidence of recurrence was observed, and overall health remained stable.

**FIGURE 9:**
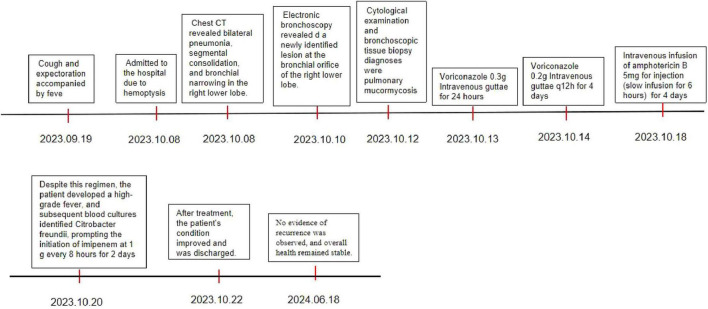
Clinical case timeline.

A summarized timeline of events has also been provided (Figure 9).

## Discussion

Mucormycosis is a life-threatening vascular-invasive fungal infection, the clinical identification of which remains difficult due to non-specific manifestations and limited diagnostic clarity of ancillary investigations.

Diagnostic approaches include fungal culture, direct smear, histopathological biopsy, and molecular methods (3). While fungal culture exhibits high specificity, its sensitivity remains below 50% (4). Quantitative polymerase chain reaction (PCR), based on serum or tissue samples, has emerged as a molecular diagnostic tool targeting prevalent Mucorales species, demonstrating sensitivity exceeding 90% and specificity above 95%. Mucorales PCR enables the detection of circulating fungal DNA, supporting diagnosis and permitting therapeutic monitoring throughout treatment (5). Matrix-assisted laser desorption/ionization time-of-flight mass spectrometry offers precise mold identification from culture isolates (6). Additionally, blood metagenomic next-generation sequencing shows potential for early detection of invasive mold infections directly from blood specimens (7), though high costs, technical complexity, and limited clinical adoption currently restrict its routine application.

Among diagnostic tools, cytological evaluation and histopathological biopsy represent the most direct and cost-effective options. Histopathology remains more commonly employed, whereas cytological diagnosis of mucormycosis is infrequent and diagnostically demanding.

Therefore, this analysis focuses on comparing cytological examination with histopathological biopsy to evaluate their respective roles and diagnostic value in the identification of mucormycosis.

In conventional cytological smears, the Pap-stained fungal elements appear red, broad, ribbon-like, folded, and undulant, resembling wrinkled kelp. The bacterial cell wall exhibits homogeneous red staining without clear zones, with hyphal branching typically forming near-right angles (∼90°) (8). In contrast, cytological examination of alveolar lavage fluid reveals more distinct hyphal structures, often twisted, folded, and exhibiting irregular branching patterns at approximately 90° angles (9).

Cell block preparations and tissue biopsy histopathology offer greater diagnostic clarity for fungal infections, including evidence of vascular infiltration (3). The hyphae typically measure 5–20 μm in width, possess thin walls, display irregular, band-like morphology with minimal septation (pauciseptate), and exhibit erratic branching lacking consistent angular orientation. Cross-sections frequently present a cystic profile, with spores rarely visualized. HE staining delineates only the hyphal wall, omitting internal structures. In contrast, GMS and PAS staining distinctly mark the fungal wall as black-brown and purple, respectively, enhancing morphological delineation.

For cytological differentiation, mucormycosis must be distinguished from fungal pathogens such as *Aspergillus, Candida*, and *Cryptococcus*. *Candida* exhibits slender (2–4 μm), beaded hyphae; *Aspergillus* features uniformly thick (5–10 μm) hyphae with dichotomous, acute-angle branching; and *Cryptococcus* appears as round, unbranched yeast-like cells.

In summary, cytological analysis offers a rapid and dependable adjunct to conventional smear-based evaluation of respiratory specimens for mucormycosis detection, despite its limited sensitivity. Liquid-based cytology enhances diagnostic yield by providing a cleaner background and more defined three-dimensional cellular structures, enabling improved morphological interpretation (10). It serves as a non-invasive and time-efficient alternative to histopathological examination, demonstrating comparable sensitivity (75.9%). In the present case, cell block preparations and histopathological biopsy revealed a relatively flattened morphological profile of mucormycosis, while liquid-based cytology presented a more compact, solid pattern. Both methods exhibited superior diagnostic performance compared to traditional smears, with added advantages of being non-invasive and expeditious.

Management of mucormycosis relies primarily on antifungal therapy (e.g., amphotericin B), surgical intervention, and adjunctive measures including acid-base balance correction and fluid resuscitation. The disease carries a high mortality rate, and timely diagnosis combined with early clinical intervention has a direct impact on patient outcomes.

## Conclusion

In this study, a case of pulmonary mucormycosis is diagnosed utilizing conventional cytology smear, liquid-based cytology, and cytological paraffin blocks. Among these, liquid-based cytology and paraffin block preparation offer a less invasive and more expedient alternative to conventional diagnostic approaches. Their diagnostic accuracy for mucormycosis proves comparable to that of histopathological examination and mucorales PCR, supporting their application in early-stage detection and prompt therapeutic intervention.

## Data Availability

The original contributions presented in this study are included in this article/supplementary material, further inquiries can be directed to the corresponding author.
